# Characterization of the mycovirus Aspergillus sulphureus partitivirus 1

**DOI:** 10.1007/s00705-025-06333-2

**Published:** 2025-06-03

**Authors:** Seiji Buma, Syun-ichi Urayama, Kenji Tomita, Sayoko Oiki, Shigeru Okada, Akihiro Ninomiya

**Affiliations:** 1 Graduate School of Agricultural and Life Sciences, The University of Tokyo, 1-1-1 Yayoi, Bunkyo-ku, Tokyo, 113-8657 Japan; 2 Faculty of Life and Environmental Sciences, University of Tsukuba, 1-1-1 Tennodai, Tsukuba, Ibaraki 305-8577 Japan; 3 Microbiology Research Center for Sustainability, University of Tsukuba, 1-1-1 Tennodai, Tsukuba, Ibaraki 305-8577 Japan; 4 Department of Fungal Infection, National Institute of Infectious Diseases, 1-23-1 Toyama, Shinjuku-ku, Tokyo, 162-8640 Japan

## Abstract

Fungi are frequently infected with viruses called mycoviruses. Some mycoviruses have shown potential as biocontrol agents because they can weaken the virulence of fungal plant pathogens. Intensive research has been conducted on the diversity of viruses infecting plant pathogens and the functions of such viruses. In contrast, the diversity of viruses infecting fungi that are not plant pathogens is less well understood. We previously identified a mycovirus of the family *Partitiviridae*, Aspergillus sulphureus partitivirus 1 (AsuPV1), from an *Aspergillus sulphureus* strain isolated from fermented dried bonito. In the present study, we conducted a detailed phylogenetic analysis of AsuPV1 and determined its molecular characteristics. The phylogenetic analysis revealed that AsuPV1 belongs to the proposed subgroup I within the genus *Gammapartitivirus*. Virus particles of AsuPV1 were isolated by density gradient centrifugation, and molecular analyses indicated that each of its three genome segments is encapsulated in capsids composed of the putative viral coat protein. AsuPV1 is the second virus with a tri-segmented genome to be found in a member of *Gammapartitivirus* subgroup I, after Penicillium stoloniferum virus F. A phenotypic comparison between the parental *A*. *sulphureus* strain and isogenic virus-free isolates indicated that AsuPV1 enhances conidium production in its host. This study provides insight into the diversity and molecular characteristics of partitiviruses with a tri-segmented genome.

**Supplementary Information:**

The online version contains supplementary material available at 10.1007/s00705-025-06333-2.

## Introduction

Mycoviruses are viruses that infect fungi. Most known mycoviruses are RNA viruses without reverse transcriptases, although several RNA viruses with reverse transcriptases and DNA viruses have been reported [1, 2]. Mycoviruses rarely kill their hosts, and they are transferred vertically via spore formation and horizontally via hyphal anastomosis [2]. Because of these characteristics, they are believed to be symbiotic with their hosts. Mycoviruses can reduce the virulence of phytopathogenic fungi, which has led to a search for viruses from phytopathogenic fungi, and analysis of viral effects on hosts [3]. However, our understanding of the diversity of viruses infecting other fungi has progressed less rapidly.

In previous work, we carried out a search for viruses from fungi of the genus *Aspergillus* isolated from fermented dried bonito, “katsuobushi”, which is a traditional Japanese food material. In an *Aspergillus sulphureus* isolate, a novel partitivirus, Aspergillus sulphureus partitivirus 1 (AsuPV1), was discovered [4]. AsuPV1 was the first mycovirus obtained from *A*. *sulphureus*. Most partitiviruses have a genome consisting of two dsRNAs [5]; however, AsuPV1 was found to have three genomic segments, and phylogenetic analysis based on amino acid sequences of the RNA-dependent RNA polymerase (RdRP) revealed that AsuPV1 belongs to the genus *Gammapartitivirus* [4]. Recently, Jaccard et al. carried out a detailed phylogenetic analysis of members of the genus *Gammapartitivirus*, and proposed that this genus be divided into subgroups I, II, and III [6]. This prompted us to carry out further phylogenetic analysis of AsuPV1. We also analyzed the molecular characteristics of AsuPV1 and compared them with those of other gammapartitiviruses.

In this study, we found that AsuPV1 belongs to *Gammapartitivirus* subgroup I. AsuPV1 is the second gammapartitivirus with three genomic segments to be found in subgroup I, after the virus that is phylogenetically closest to AsuPV1, Penicillium stoloniferum virus F (PsV-F) [7]. We also isolated particles of AsuPV1 by density gradient centrifugation and found that they were composed of the viral coat protein (CP) and each of the three genome segments. To investigate the effect of AsuPV1 on its host, the virus was eliminated from the host using antiviral drugs, and the phenotypes of the parental strain and the AsuPV1-free isogenic strains were then compared. The results suggested that AsuPV1 enhances conidia production in its hosts.

## Materials and methods

### Fungus

*A*. *sulphureus* strain NBRC4095 was provided by the Biological Resource Center, National Institute of Technology and Evaluation (NBRC; Tokyo, Japan). According to the information available from NBRC, this strain was isolated from katsuobushi received by the Institute for Fermentation, Osaka, in 1941, and then transferred to NBRC. For maintenance of this strain, it was cultured at 26.5°C on potato dextrose agar (PDA), and this temperature was used in the subsequent experiments.

### Phylogenetic analysis

The genome sequence of AsuPV1 (GenBank accession numbers LC832460, LC832461, and LC832462) was determined in a previous study [4]. Phylogenetic trees were constructed based on the RdRP amino acid sequences of selected partitiviruses. Multiple sequence alignments were performed using MAFFT software v7.490 [8]. Substitution models were tested, and the Q.pfam + F + R6 model was chosen using ModelFinder [9]. A maximum-likelihood tree (1000 bootstrap replicates) was constructed using IQ-TREE software v2.2.2.6 [10]. The sequences of the third segments of gammapartitiviruses were aligned using MAFFT, and pairwise identities were calculated.

### Purification of mycovirus particles

*A*. *sulphureus* was cultured on PDA. The mycelia were then cut out with the agar pieces, inoculated into 100 mL of potato dextrose broth (PDB), and incubated at 26.5°C with agitation at 150 rpm for one week. Mycelia and culture supernatant were separated by filtration using Prowipe (Daio Paper Corporation, Tokyo, Japan), and the mycelia were stored at -80°C. The frozen mycelia were crushed in buffer A (0.2 M KCl, 0.05% β-mercaptoethanol, 0.1 M sodium phosphate, pH 7.4) using a mixer (Tescom Denki, Tokyo, Japan). The homogenate was centrifuged (4°C, 9,000 *g*, 10 min), and the supernatant was centrifuged again (4°C, 12,000 *g*, 1 h). The virus was then collected by ultracentrifugation (4°C, 190,000 *g*, 1 h), and the pellet was suspended in 1 mL of buffer B (50 mM Tris-HCl, 150 mM NaCl, 5 mM EDTA, pH 7.8). The suspension was centrifuged (4°C, 15,000 *g*, 1 min), and the supernatant fraction containing virus particles was collected. The precipitate was resuspended in 500 µL of buffer B and centrifuged (4°C, 15,000 *g*, 1 min), and the supernatant was combined with the previously collected fraction. The virus particle fraction was then subjected to density gradient centrifugation using OptiPrep (Serumwerk Bernburg AG, Bernburg, Germany). Layers of buffer B containing 50, 40, 35, 30, 25, and 20% iodixanol were prepared in centrifuge bottles 30PC (Eppendorf, Hamburg, Germany). The suspension of virus particles (1 mL in total) and OptiPrep (60% iodixanol) were mixed, and 50% and 40% iodixanol layers were prepared. After ultracentrifugation (4°C, 190,000 *g*, 3 h), nine 3-mL fractions were collected from the top to the bottom. Buffer B was added to each fraction, and after centrifugation (4°C, 190,000 *g*, 1 h), the pellets were resuspended in 400 µL of sterile water.

### Agarose gel electrophoresis and SDS-PAGE

dsRNA was isolated from 100-µL aliquots of the fraction obtained by density gradient centrifugation as described previously [11, 12], and the dsRNA fractions were analyzed by 1.2% agarose gel electrophoresis. In addition, a 20-µL aliquot of each fraction obtained by density gradient centrifugation was subjected to SDS-PAGE as described previously [13]. Peptide mass fingerprinting analysis was performed by Japan Proteomics Co., LTD (Miyagi, Japan).

### Negative staining

A soft plasma etching system (SEDE-AF; Meiwafosis, Tokyo, Japan) was used to hydrophilize a copper grid with a carbon support film. A suspension of virus particles (5 µL) was placed on the grid and allowed to stand for five minutes to allow the virus particles to attach to the support membrane. The liquid was removed by absorbing it onto filter paper, and 5 µL of 1% (w/v) uranium acetate solution was immediately placed on the grid. After 3 minutes, the excess uranium solution was removed using filter paper, and the grids were air-dried for negative staining. Electron microscopy was performed using a JEM-1400Plus transmission electron microscope (JEOL Ltd., Tokyo, Japan) at an acceleration voltage of 120 kV. A standard-equipped CCD camera was used for imaging.

### Elimination of mycoviruses

Elimination of AsuPV1 was performed as reported previously [4]. The nucleoside analogs 2'-*C*-methylcytidine (2CMC; Combi-Blocks, San Diego, CA, USA) and ribavirin (Combi-Blocks) were used as antiviral drugs, and dimethyl sulfoxide (DMSO) was used as a negative control. For cultures, a combination of PDA and PDB, or a combination of yeast extract-peptone-2% sucrose agar (YPS2A; 1% yeast extract, 2% peptone, 2% sucrose, 2% agar) and yeast extract-peptone-20% sucrose medium (YPS20; 1% yeast extract, 2% peptone, 20% sucrose) was employed. Extraction of total RNA from mycelia and reverse transcription PCR (RT-PCR) were performed as reported previously [4]. The sequences of primers used for RT-PCR were 5’-TTTCATTGGAATGCGAGGCG-3’ (forward) and 5’-TTTCCGTAAGCCATCTACCC-3’ (reverse).

### Homology modeling

The SWISS-MODEL program [14] was used to perform homology modeling of the AsuPV1 CP. The structure of PsV-F CP (SMTL ID: 3es5.1) was used as a template. Figures were drawn using Geneious Prime 2024 (https://www.geneious.com) and PyMOL software [15].

### Analysis of growth rate

PDA, YPS2A, and minimal medium [16] containing 2% (w/v) sucrose and 1.5% agar (MMS2A) were used for culture. One hundred spores suspended in 0.2 µL of 0.05% Tween 20 were dropped at the center of the agar plate and incubated. Colony diameters were measured every 12 hours for 1 week. The average growth rate (36–108 h) was determined for each culture. Three independently obtained AsuPV1-free isolates were used for this experiment. The parental strain and the three AsuPV1-free isolates were each cultured in triplicate. For each AsuPV1-free isolate, the mean value of the growth rates in the three replicate cultures was taken as the growth rate of that isolate, and the mean value of the growth rates of the three AsuPV1-free isolates was taken as the growth rate of the AsuPV1-free strain. The significance of differences in growth rates between the parental strain and the AsuPV1-free strain was evaluated using Welch's *t*-test.

### Analysis of conidia production

One hundred spores suspended in 0.2 µL of 0.05% Tween 20 were dropped at the centers of PDA plates and incubated. Six days after inoculation, 5 mL of 0.05% Tween 20 was added to each plate, and the fungal colony was scrubbed off using a cell spreader. The conidial suspension was filtered through a cell strainer, pluriStrainer 40 µm (pluriSelect Life Science, Leipzig, Germany). The density of conidia in the suspension (5 mL) was determined using an improved Neubauer's counting chamber (Watson, Tokyo, Japan). Three independently obtained AsuPV1-free isolates were used for this experiment. The parental strain and the three AsuPV1-free isolates were cultured in triplicate. For each AsuPV1-free isolate, the mean value of the conidia densities in its three cultures was taken as the conidia density of that isolate. Then, the mean value of the conidia densities of the three AsuPV1-free isolates was taken as the conidia density of the AsuPV1-free strain. The significance of differences in conidia densities between the parental strain and the AsuPV1-free strain was evaluated using Student's *t*-test.

## Results

### Phylogenetic analysis

**Fig. 1:**
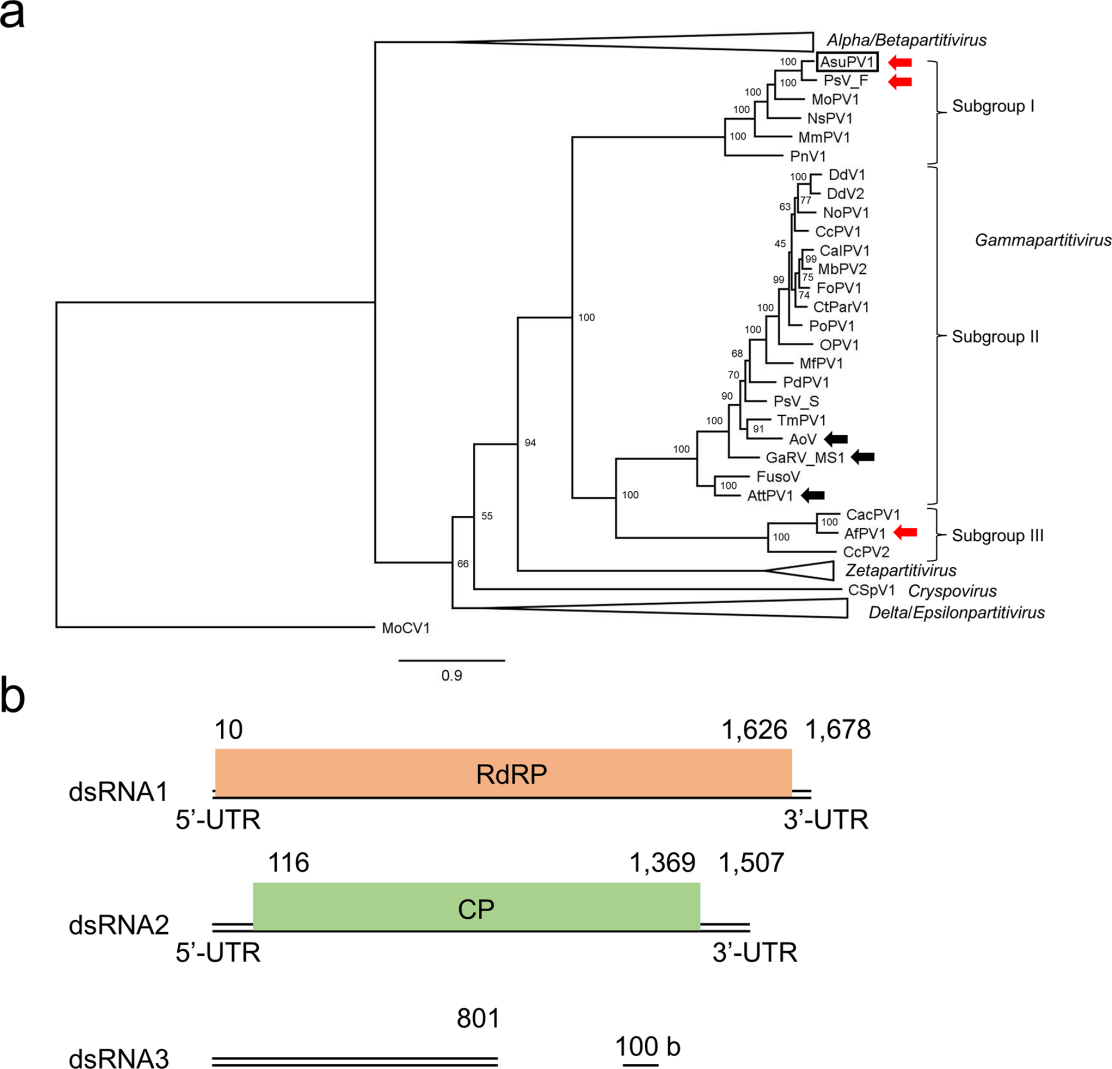
Phylogenetic analysis and genomic organization of Aspergillus sulphureus partitivirus 1 (AsuPV1). (**a**) A maximum-likelihood tree of selected members of the family *Partitiviridae* based on RNA-dependent RNA polymerase (RdRP) amino acid sequences. AsuPV1 is shown enclosed in a rectangle. The black or red arrows indicate viruses with tri-segmented genomes. The other gammapartitiviruses in the tree have bi-segmented genomes. The red arrows indicate that encapsidation of each genomic segment of the indicated virus was confirmed experimentally. The numbers on the branches are bootstrap percentages based on 1000 replicates. MoCV1, of the family *Chrysoviridae*, was used as an outgroup. The full names and accession numbers of the viruses used to build the tree are shown in Supplementary Table S1. For the unabbreviated tree, see Supplementary Figure S7. (**b**) Genomic organization of AsuPV1. RdRP, RNA-dependent RNA polymerase; CP, coat protein

We performed a phylogenetic analysis of 75 partitiviruses, including AsuPV1, based on RdRP amino acid sequences to determine the subgroup to which AsuPV1 belongs. The results showed that AsuPV1 is included in subgroup I (Fig. 1a), as is Penicillium stoloniferum virus F (PsV-F), the phylogenetically closest relative of AsuPV1. The genome of AsuPV1 consists of three dsRNAs (Fig. 1b). While most partitiviruses have a bi-segmented genome, some (see Fig. 1a) have additional segments [5]. Subgroups I, II, and III all include some viruses with tri-segmented genomes and some with bi-segmented genomes. PsV-F, belonging to subgroup I, has a tri-segmented genome. The third segment of PsV-F (677 bp; GenBank accession number AY738338) encodes a protein of 54 amino acids with unknown function [7]. In subgroup II, Aspergillus ochraceus virus (AoV), Gremmeniella abietina RNA virus MS1 (GaRV-MS1), and Alternaria tenuissima partitivirus 1 (AttPV1) also have tri-segmented genomes. The third segment of AoV (1,220 bp; GenBank accession number EU118279) encodes a protein of 293 amino acids with unknown function [17, 18], that of GaRV-MS1 (1,186 bp; GenBank accession number AY089995) encodes a protein of 237 amino acids [19], and that of AttPV1 (1,537 bp; GenBank accession number MT648468) encodes a putative CP of 432 amino acids [20]. Aspergillus flavus partitivirus 1 (AfPV1), belonging to subgroup III, also has a tri-segmented genome. The third segment of AfPV1 (1,186 bp; GenBank accession number MK344770) encodes a protein of 280 amino acids with unknown function [21]. The sequences of the third segments of these tri-segmented gammapartitiviruses (AsuPV1, PsV-F, AoV, GaRV-MS1, AttPV1, and AfPV1) were aligned, and pairwise identity values were calculated (Supplementary Table S2). The third segments of AsuPV1 and PsV-F were found to be 38.66% identical, while the other identity values were lower (19.49–33.31%).

**Fig. 2:**
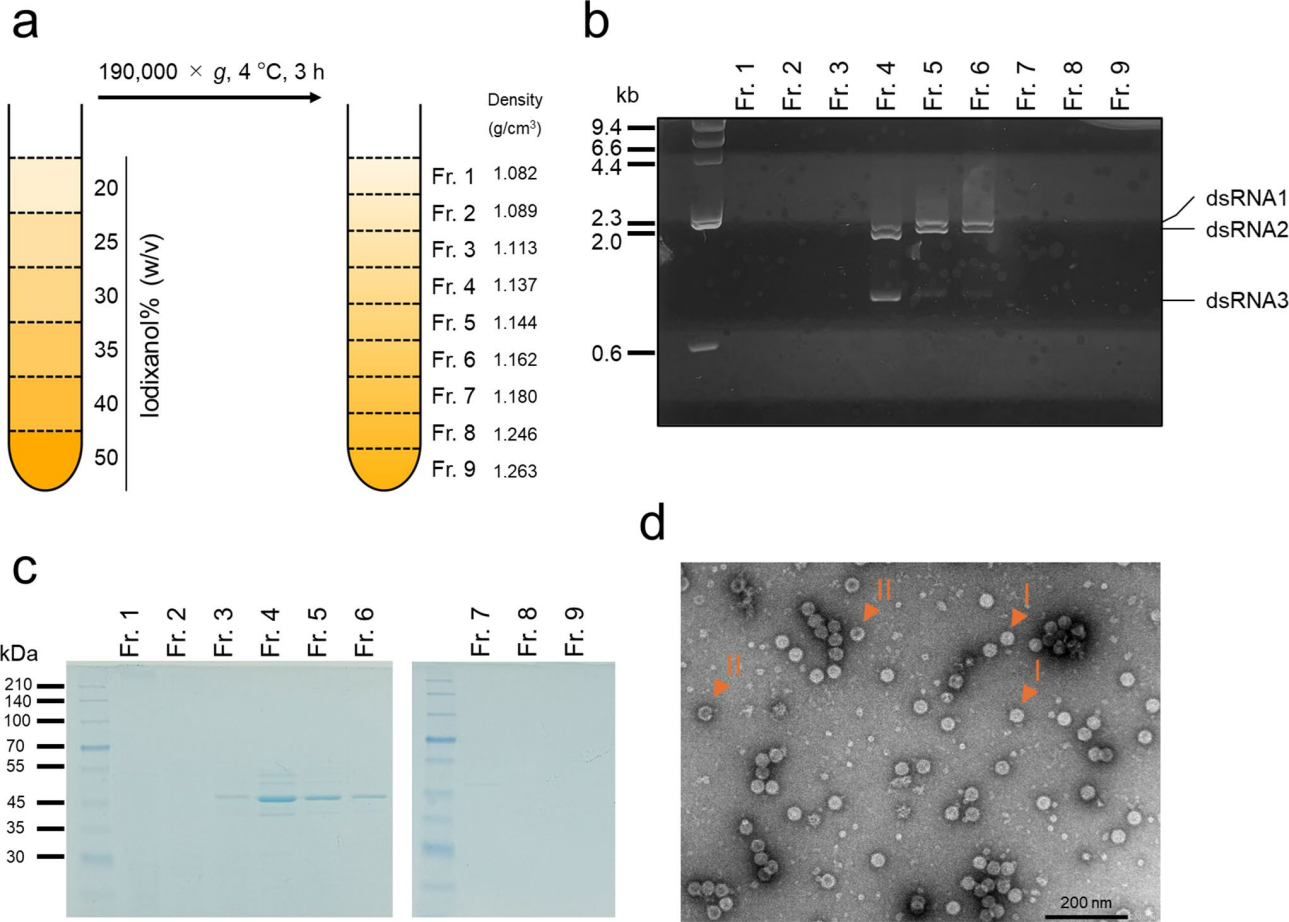
(**a**) Details of iodixanol density gradient centrifugation. (**b**) Electrophoresis of dsRNA extracted from each fraction obtained by density gradient centrifugation. (**c**) SDS-PAGE of proteins extracted from each fraction obtained by density gradient centrifugation. (**d**) Electron micrograph of negatively stained particles from fraction 6. The particles with and without genomic RNA are indicated by the Roman numerals I and II, respectively

**Fig. 3:**
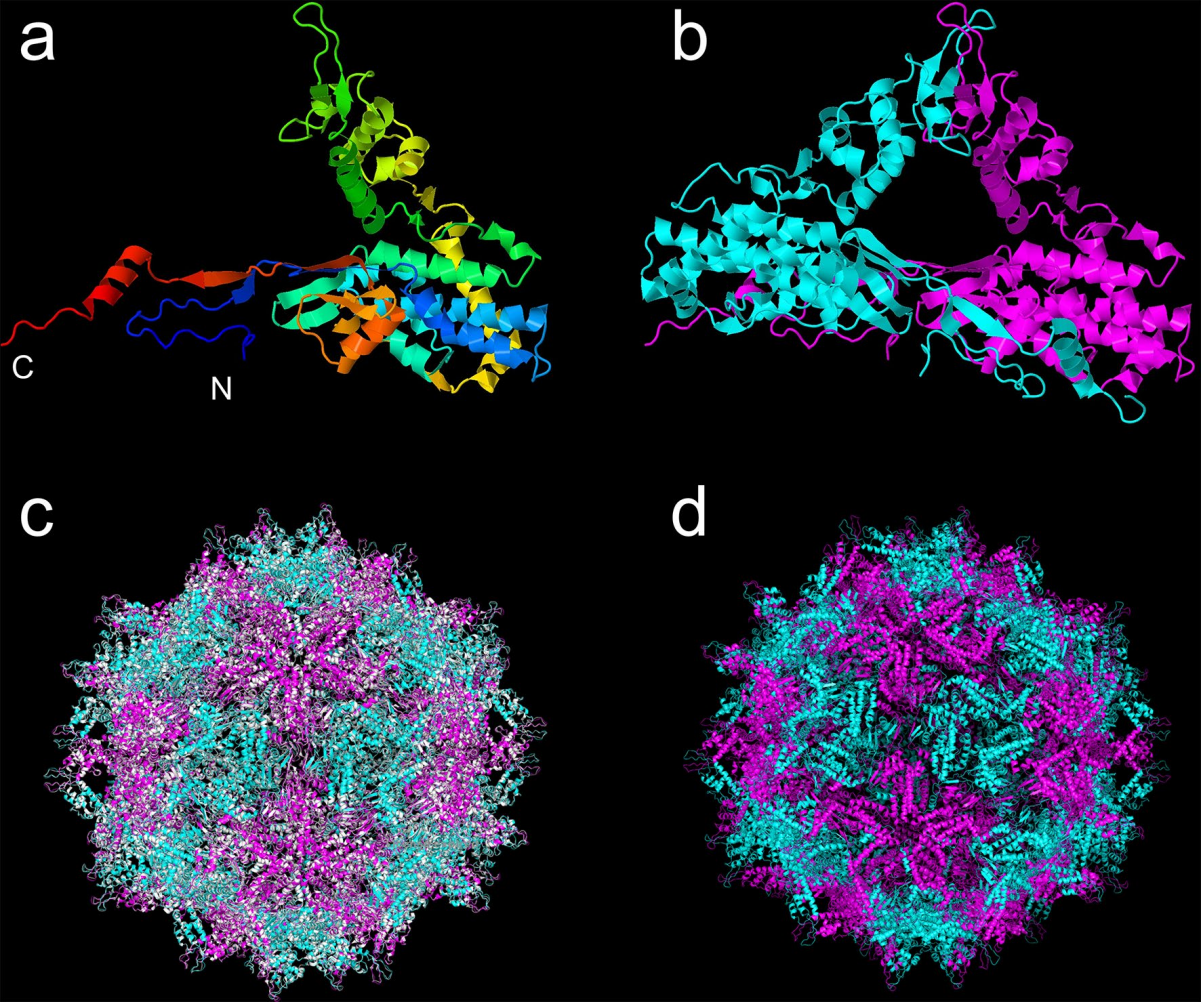
Quaternary structure of the CP of AsuPV1 predicted using SWISS-MODEL. (**a**) A CP monomer showing a reclining-V shape. The letters N and C indicate the N- and C-termini of the CP, respectively. (**b**) A CP dimer with the subunits colored in cyan and magenta. (**c**) Superimposition of three-dimensional structures of the Penicillium stoloniferum virus F (PsV-F) capsid (white) and the predicted AsuPV1 capsid (cyan and magenta). (**d**) Predicted three-dimensional structure of the AsuPV1 capsid

**Fig. 4:**
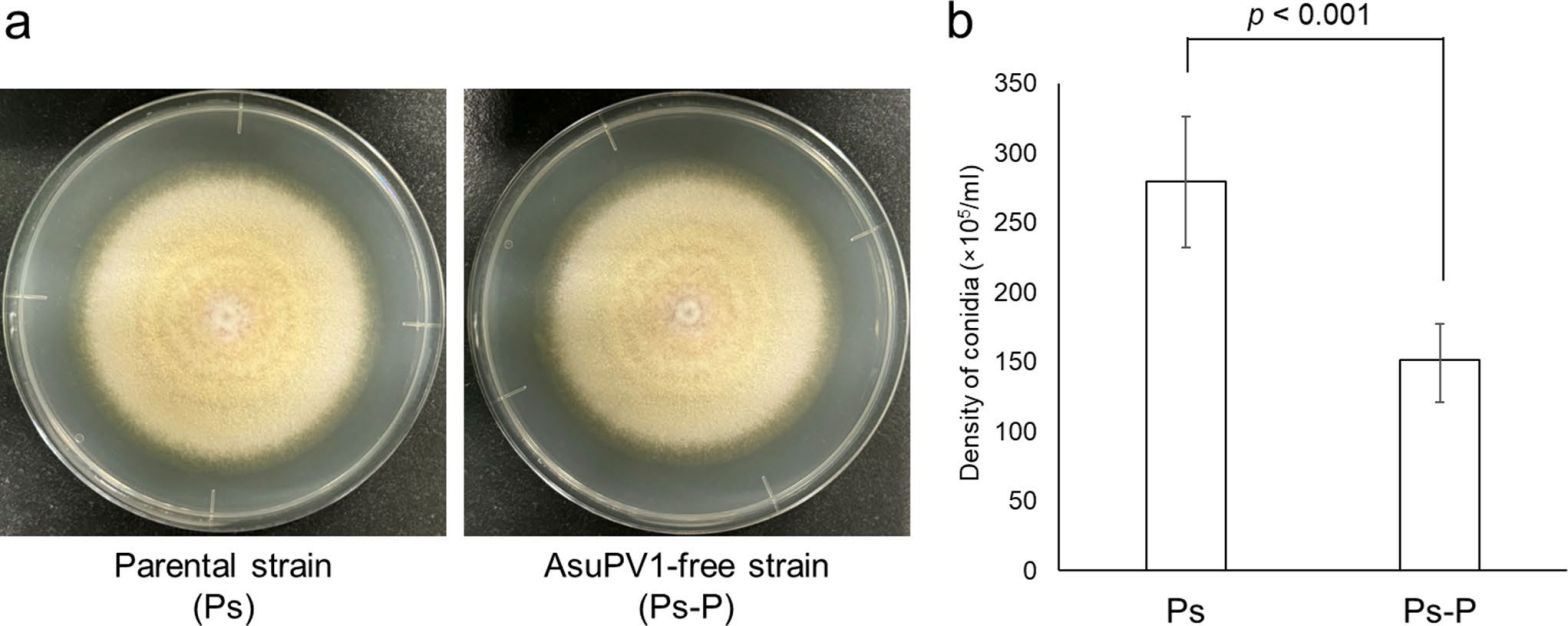
(**a**) Colony morphology of the parental *Aspergillus sulphureus* strain NBRC4095 (Ps) and the isogenic strain without AsuPV1 (Ps-P) grown on potato dextrose agar (PDA) plates for 6 days. (**b**) Comparison of conidia formation between Ps and Ps-P. After culture on PDA for 6 days, the conidia were suspended in 5 mL of 0.05% Tween 20, and the density of conidia was calculated. Three independently obtained strains of Ps-P were used. Each strain was cultured in triplicate. Error bars indicate standard deviation. Significance was tested using Student’s *t*-test

### Composition of virus particles

In order to study their composition, we isolated virus particles of AsuPV1 from its host fungus *A*. *sulphureus*. In previous studies, cesium chloride [7, 22, 23] or sucrose [21, 24–33] was used as a density gradient medium for purification of partitivirus particles, but in the present study, we decided to use iodixanol in light of a recent study in which the empty capsid of a partitivirus was successfully isolated by density gradient centrifugation using iodixanol [34]. A cell homogenate of *A*. *sulphureus* NBRC4095 was fractionated by centrifugation and density gradient ultracentrifugation to obtain nine fractions (fractions 1–9; Fig. 2a). Fractions 1–9 were purified to obtain dsRNAs, which were subjected to electrophoresis, which showed that fractions 4, 5, and 6 contained dsRNA1, dsRNA2, and dsRNA3 (Fig. 2b). Fractions 1–9 were also analyzed by SDS-PAGE, which showed that fractions 4, 5, and 6 contained a protein yielding an intense band around 45 kDa (Fig. 2c), which was consistent with the predicted molecular weight of the putative CP based on its amino acid sequence (47 kDa). This band was cut out and subjected to peptide mass fingerprinting analysis. Mascot analysis [35] revealed that 36 peptide fragments matched the amino acid sequence of the putative CP (coverage, 48%; Mascot score, 919) (Supplementary Fig. S1), confirming that this band corresponded to the putative CP. Together, these results indicated that fractions 4, 5, and 6 contained viral particles. Because the particles in fractions 4 and 5 appeared to be partially aggregated, particles from fraction 6 were negatively stained and examined by electron microscopy (Fig. 2d). The particle size was estimated to be about 40 nm, which is consistent with the typical size of a gammapartitivirus particle (25–43 nm) [5]. These results showed that the putative CP is a structural protein and that each of the genome segments is encapsidated.

To confirm that the particles observed in Fig. 2d were virus particles of AsuPV1, we compared the host *A*. *sulphureus* NBRC4095 with an isogenic virus-free isolate. To remove AsuPV1, the host was treated with the antiviral drugs 2CMC and ribavirin using two different agar media (YPS2A and PDA). While no virus-free strains were obtained using YPS2A, with PDA, the elimination rate of AsuPV1 was 95% (19 virus-free isolates/20 isolates) (Supplementary Fig. S2). Hereinafter, we refer to the parental strain as "Ps", and the isogenic strains without AsuPV1 as "Ps-P". Mycelia (10 g each) of the strains Ps and Ps-P were homogenized, and each homogenate was fractionated by density gradient centrifugation as described above. The resulting fractions were subjected to SDS-PAGE (Supplementary Fig. S3a and b), and bands corresponding to the CP were observed in fractions 3, 4, 5, and 6 from Ps, but not in the fractions from Ps-P. Furthermore, no putative virus particles were observed in the microscopic fields of fractions 4, 5, and 6 from Ps-P (Supplementary Fig. S3c). These results support the conclusion that the putative virus particles observed in strain Ps were virus particles of AsuPV1.

AsuPV1 particles were isolated from *A*. *sulphureus* NBRC4095 (Ps) using density gradient centrifugation with iodixanol as the density medium. Iodixanol is typically used for separation of cell organelles or membrane vesicles, and it is also often used for purification of viruses [36, 37]. Although the empty capsid composed of recombinant CP of a partitivirus has been purified by iodixanol density gradient centrifugation [34], no partitivirus virions have yet been purified using this method. In light of the findings of the present study, iodixanol may be added to the list of density medium options for purification of partitivirus virions.

### Three-dimensional structure of AsuPV1 capsid

To predict the three-dimensional structure of the CP of AsuPV1, homology modeling was performed using SWISS-MODEL [14]. Because the CP of AsuPV1 showed the highest similarity to that of PsV-F (identity, 71%), the structure of PsV-F was used as a template (GMQE, 0.79; QMEDisCo global score, 0.80 ± 0.05). The three-dimensional structure of the PsV-F capsid has been determined previously by X-ray crystallography [38]. In the PsV-F capsid, the CP molecules form homodimers. The CP of AsuPV1 was predicted to have a reclining-V shape and form homodimers like those of PsV-F (Fig. 3a and b). Furthermore, cryo-electron microscopy analysis of PsV-F particle has revealed that the PsV-F capsid consists of 60 equivalent CP dimers arranged with icosahedral symmetry [38]. Therefore, the predicted CP dimer structure of AsuPV1 was superimposed onto the 60 PsV-F capsid proteins to make a three-dimensional model of the entire AsuPV1 capsid (Fig. 3c and d). In this model, the structures of the CP dimers of AsuPV1 and PsV-F were highly similar, with a root-mean-square deviation value of 0.132. Like the PsV-F capsid, the AsuPV1 capsid was predicted to form an icosahedral structure consisting of 60 CP dimers without structural constraints.

### Effect of AsuPV1 on its host

Three independently obtained virus-free strains were used for the following analyses. First, the effect of AsuPV1 on the growth of the host was evaluated using three types of agar media (PDA, YPS2A, and MMS2A). No difference was observed between the growth rates of the parental strain Ps and the AsuPV1-free strain (Ps-P) in cultures on the above-mentioned media (Supplementary Figs. S5 and S6), indicating that AsuPV1 has little effect on the radial growth of the host. Because *A*. *sulphureus* seemed to form more conidia on PDA, the conidium production of Ps on PDA was compared with that of Ps-P. No significant difference was observed between the macroscopic phenotypes of Ps and Ps-P grown on PDA (Fig. 4A). However, strain Ps was found to form more conidia than Ps-P (Fig. 4B), suggesting that conidium production in *A*. *sulphureus* is enhanced by AsuPV1.

## Discussion

Most known partitiviruses have two genome segments; however, some have three. To the best of our knowledge, AsuPV1 is the second virus belonging to *Gammapartitivirus* subgroup I to be shown to have three segments, after PsV-F. The genome organization of mycoviruses is usually inferred on the basis of similarities in the terminal sequences of each segment. It is rare for researchers to isolate a virion and confirm that the particles contain each segment. In addition to AsuPV1, encapsidation of each genomic segment has been experimentally confirmed for PsV-F and AfPV1 (Fig. 1). Although the association between virus particles and dsRNAs has been reported for some partitiviruses [23, 33], this is only the second case, after PsV-F, where viral particles of a virus belonging to *Gammapartitivirus* subgroup I have been isolated and the number of segments confirmed. In the case of PsV-F, it was reported previously that each of the three segments can be incorporated into the capsid [7]. As described above, AsuPV1 shares multiple features with the phylogenetically closest virus, PsV-F. In our density gradient centrifugation experiment, the relative density ratios of bands of dsRNA1, dsRNA2, and dsRNA3 differed in fractions 4, 5, and 6 (Fig. 2b), suggesting that each of the three segments is encapsidated separately. A similar phenomenon has been observed with Magnaporthe oryzae chrysovirus 1 [39].

The genera *Alphapartitivirus* [4, 31], *Betapartitivirus* [40], and *Gammapartitivirus* [4, 7, 17, 19–21], and the proposed genus "*Zetapartitivirus*" [26, 41, 42] all include partitiviruses with three genome segments (Supplementary Fig. S7). dsRNA3 of Rosellinia necatrix partitivirus 2 (RnPV2), which belongs to the genus *Alphapartitivirus*, is thought to have resulted from a deletion of part of the dsRNA1 sequence [31]. In the case of AttPV1 [20], which belongs to *Gammapartitivirus* subgroup II, the pairwise identity of dsRNA2 and dsRNA3 is 79.79%. This suggests that these dsRNAs share the same origin. In the case of AsuPV1, dsRNA3 is 32.29% identical to dsRNA1 and 24.90% to dsRNA2. It is therefore unlikely that dsRNA3 arose from dsRNA1 or dsRNA2. To test the possibility of horizontal transmission of dsRNA3 from other organisms, dsRNA3 sequences were subjected to BLASTn analysis, but no homologous sequences were found.

The three-dimensional structure of the AsuPV1 capsid was predicted by homology modeling. In the model, the AsuPV1 CP forms a homodimer, and 60 equivalent homodimers form the capsid, as has been demonstrated for PsV-F. The PsV-F CP has a disordered region at its N-terminus, which contains several basic residues (Lys and Arg). In a previous study, cryo-electron microscopy analysis indicated that the disordered N-terminal region interacts with genomic dsRNA inside the capsid [38]. The N-terminal region of the AsuPV1 CP also contains several basic residues; the proportion of Lys or Arg residues at amino acid positions 1–40 is 17.5%, while that in the full-length AsuPV1 CP is 10.8%. This suggests that the N-terminal disordered region of the AsuPV1 CP also interacts with dsRNA.

In this study, AsuPV1 was eliminated from *A*. *sulphureus* strain NBRC4095 using the antiviral drugs 2CMC and ribavirin. Under the conditions used in this study, when PDA was used as the culture medium, 19 of the 20 isolates obtained were virus-free; however, when YPS2A was used, no virus-free isolates were obtained. This suggests that the efficiency of virus elimination depends on the composition of the culture medium. Although further study is needed to understand the effect of culture media on virus elimination, trying multiple types of media could be effective for achieving successful virus elimination. To examine the potential use of 2CMC and ribavirin along with protoplasts for virus elimination, the minimum inhibitory concentrations (MICs) of these drugs against *A*. *sulphureus* NBRC4095 were determined (Supplementary Table S3; for methods, see Supplementary Material). The MIC values of 2CMC and ribavirin in two types of medium, yeast extract-peptone-2% sucrose medium (YPS2) and PDB, were higher than 2 mg/mL. Considering the concentration of 2CMC and ribavirin used for virus elimination (0.2 mg/mL and 0.1 mg/mL, respectively), it was supposed that 2CMC and ribavirin could be used for protoplasts of *A*. *sulphureus*.

Classically, partitiviruses were believed to have little effect on their hosts; however, it has been reported recently that they can affect the temperature sensitivity and competitive ability of their hosts [43]. A betapartitivirus, Sclerotinia sclerotiorum partitivirus 1, has been shown to enhance conidium production in hosts [22]. Several gammapartitiviruses are also known to affect conidiation. While Colletotrichum alienum partitivirus 1 enhances conidiation in its hosts [27], Fusarium oxysporum partitivirus 1 [44] and Metarhizium majus partitivirus 1 [45] reduce conidium production. To the best of our knowledge, AsuPV1 is the second gammapartitivirus that activates host conidiation. However, there remains a possibility that other mycoviruses that we did not detect are involved in activation of host conidiation. Further study is needed to determine the molecular mechanism underlying activation of conidium production.

## Conclusion

Phylogenetic analysis based on the RdRP amino acid sequences revealed that AsuPV1 is the second tri-segmented gammapartitivirus belonging to the proposed subgroup I. A cell homogenate of the host was fractionated by density gradient ultracentrifugation, and virus particles were obtained. Negative staining revealed that AsuPV1 forms particles with a size of about 40 nm. The composition of the particles was analyzed by SDS-PAGE, Mascot search, and agarose gel electrophoresis, which indicated that each of the three segments of AsuPV1 is encapsulated in capsids composed of the putative CP. Furthermore, the phenotypes of the parental *A*. *sulphureus* strain and the AsuPV1-free isogenic strains were compared, providing evidence that AsuPV1 promotes conidiation in its host. The results of the present study provide insight into the diversity and molecular characteristics of partitiviruses with a tri-segmented genome.

## Electronic Supplementary Material

Below is the link to the electronic supplementary material

Supplementary Material 1
