# Supplementary material for: Characterization of the mycovirus Aspergillus sulphureus partitivirus 1
Source: Arch Virol. 2025 Jun 3;170(7):147. doi: 10.1007/s00705-025-06333-2 (PMC12130121; doi:10.1007/s00705-025-06333-2)
Supplement: Supplementary file 1 — Supplementary Material 1 [file 705_2025_6333_MOESM1_ESM.docx]

**Characterization of the mycovirus Aspergillus sulphureus partitivirus 1**

Seiji Buma,^1^ Syun-ichi Urayama,^2, 3^ Kenji Tomita,^1^ Sayoko Oiki^4^, Shigeru Okada,^1^ Akihiro Ninomiya^1*^

^1^Graduate School of Agricultural and Life Sciences, the University of Tokyo, 1-1-1 Yayoi, Bunkyo-ku, Tokyo 113-8657, Japan.

^2^Faculty of Life and Environmental Sciences, University of Tsukuba, 1-1-1 Tennodai, Tsukuba, Ibaraki 305-8577, Japan.

^3^Microbiology Research Center for Sustainability, University of Tsukuba, 1-1-1 Tennodai, Tsukuba, Ibaraki 305-8577, Japan.

^4^Department of Fungal Infection, National Institute of Infectious Diseases, 1-23-1 Toyama, Shinjuku-ku, Tokyo 162-8640, Japan

*Corresponding author

ninomiya-akihiro@g.ecc.u-tokyo.ac.jp

**Supplementary figures**

Figure S1. Peptide fragments detected in the peptide mass finger printing analysis.

Figure S2. Elimination rate of AsuPV1 from *Aspergillus sulphureus* NBRC4095.

Figure S3. Fractionation of homogenate of the parental *Aspergillus sulphureus* strain NBRC4095 (Ps), and the isogenic strain without AsuPV1 (Ps-P).

Figure S4. Agarose gel electrophoresis of total RNA and double-stranded RNA fractions from the parental strain Ps and three independently obtained AsuPV1-free isolates (Ps-P-1, 2, and 3).

Figure S5. Comparison of colony diameter between the parental strain (Ps) and the isogenic strains without AsuPV1 (Ps-P) grown on minimal agar medium containing 2% sucrose (MMS2A), YPS2A, and PDA.

Figure S6. Comparison of average radial growth rates between Ps and Ps-P grown on MMS2A, YPS2A, and PDA.

Figure S7. A maximum likelihood tree of selected members of the family *Partitiviridae* based on RNA-dependent RNA polymerase (RdRP) amino acid sequences.

**Supplementary tables**

Table S1. List of partitiviruses used for phylogenetic analyses.

Table S2. Pairwise identities of the third genomic segments of gammapartitiviruses with a tri-segmented genome.

Table S3. Minimum inhibitory concentrations of 2'-*C*-methylcytidine (2CMC) and ribavirin.

**Supplementary methods**

Preparation of protoplasts

Minimum inhibitory concentration (MIC) test using conidia

MIC test using protoplasts

Figure S1. Peptide fragments detected in the peptide mass finger printing analysis. The amino acid sequence of the coat protein (CP) of Aspergillus sulphureus partitivirus 1 (AsuPV1) is shown. The matched peptides are indicated by bold letters.


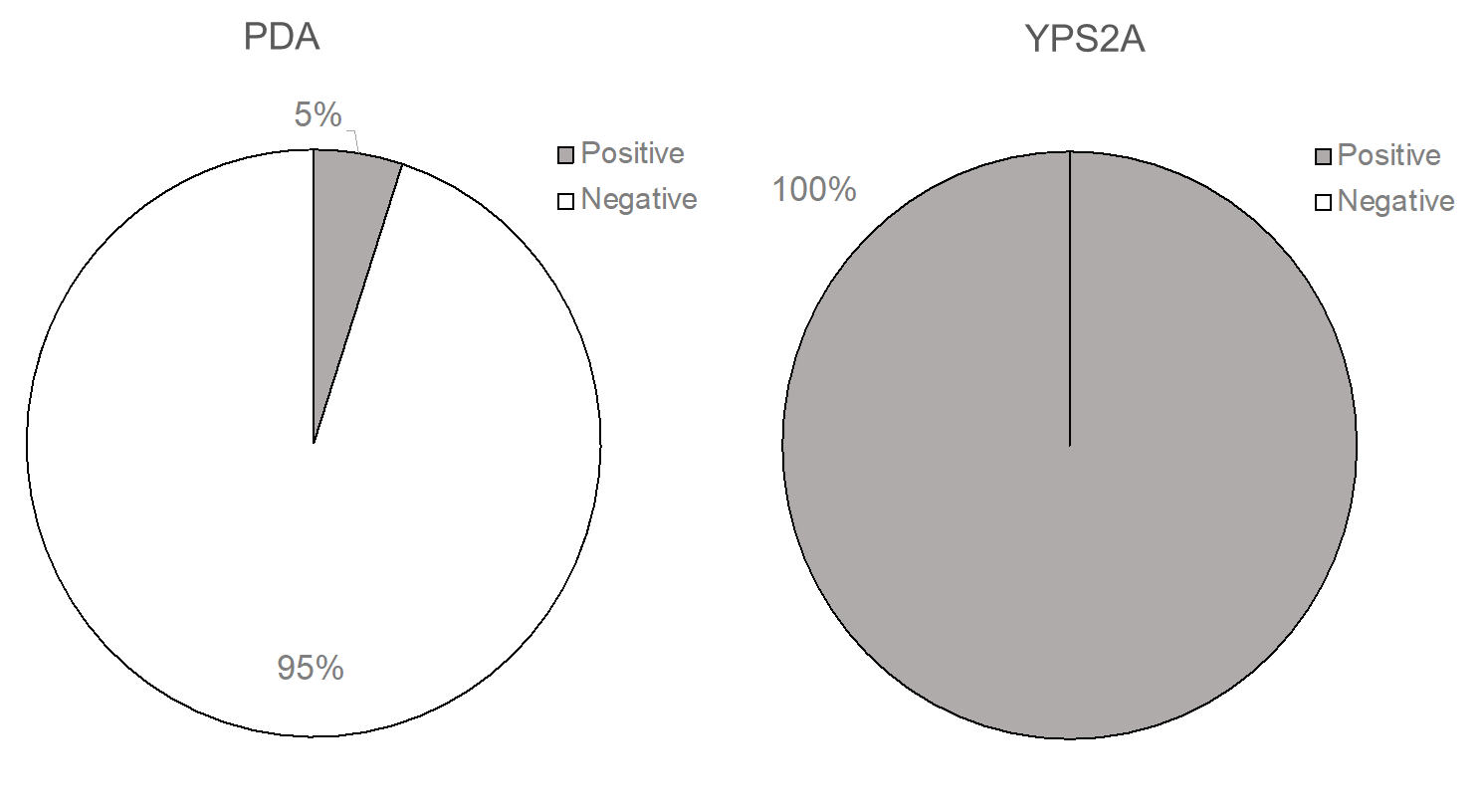


Figure S2. Elimination rate of AsuPV1 from *Aspergillus sulphureus* NBRC4095. After treatment with 2CMC and ribavirin, existence of each mycovirus was confirmed by reverse transcription (RT)-PCR using primers specific to AsuPV1. Two types of agar media [potato dextrose agar (PDA) and yeast extract-peptone-2% sucrose agar (YPS2A)] were used for culture.


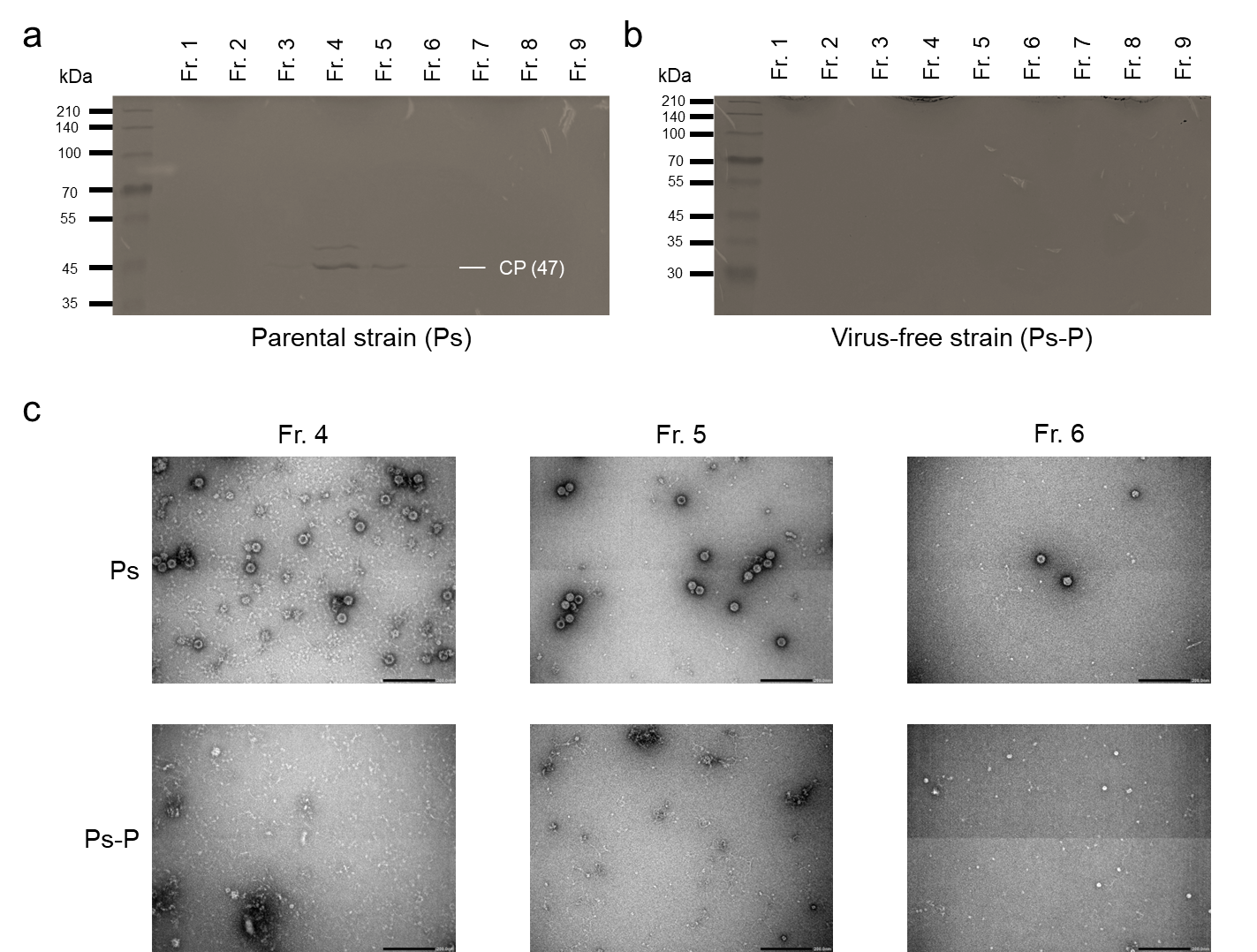


Figure S3. Fractionation of homogenate of the parental *Aspergillus sulphureus* strain NBRC4095 (Ps), and the isogenic strain without AsuPV1 (Ps-P). (a, b) SDS-PAGE of the fractions obtained by density gradient ultracentrifugation from Ps and Ps-P, respectively. (c) Fractions 4, 5, and 6 from Ps and Ps-P were observed by negative-staining. The scale bars are 200 nm.


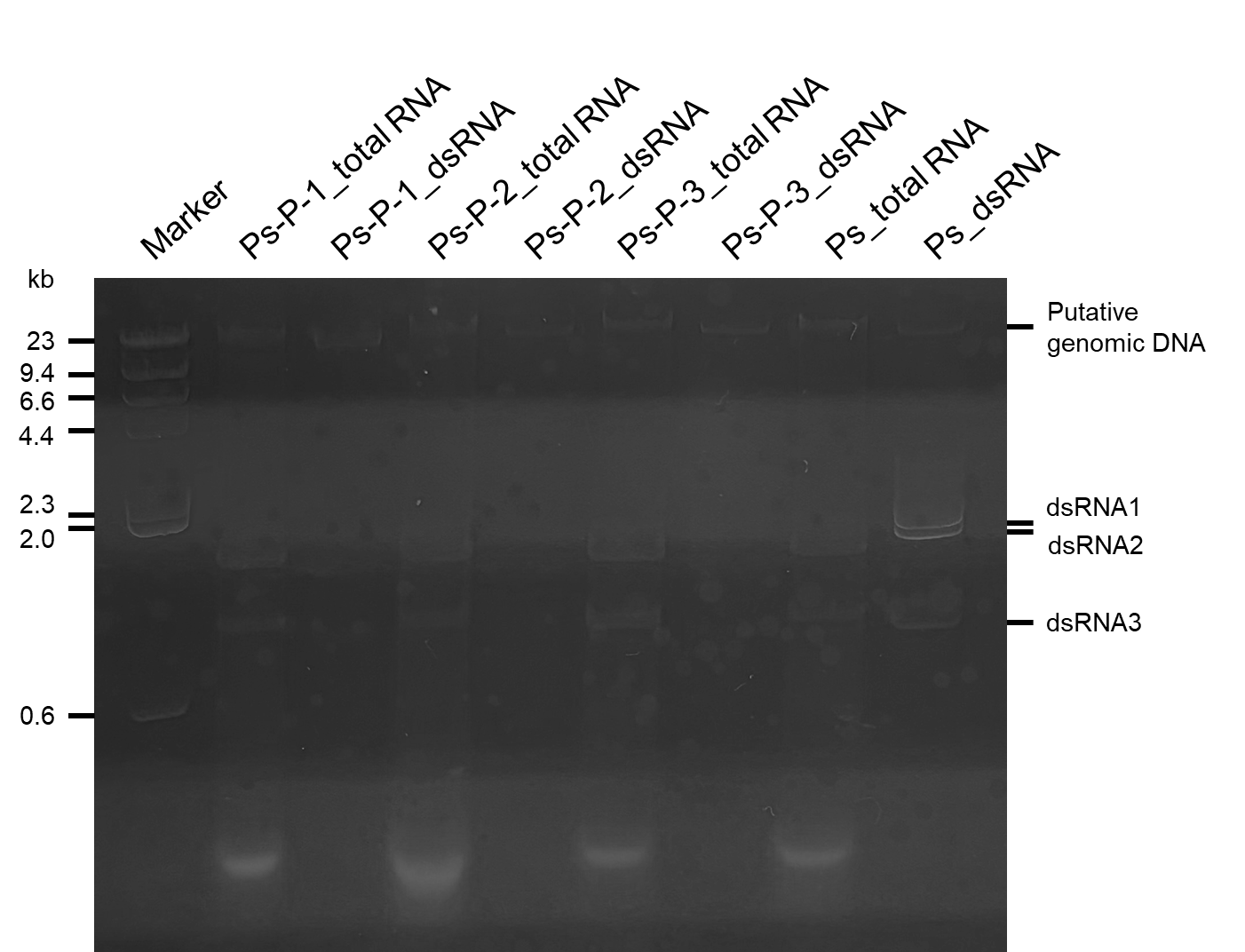


Figure S4. Agarose gel electrophoresis of total RNA and double-stranded RNA from the parental strain Ps and three independently obtained AsuPV1-free isolates (Ps-P-1, 2, and 3).


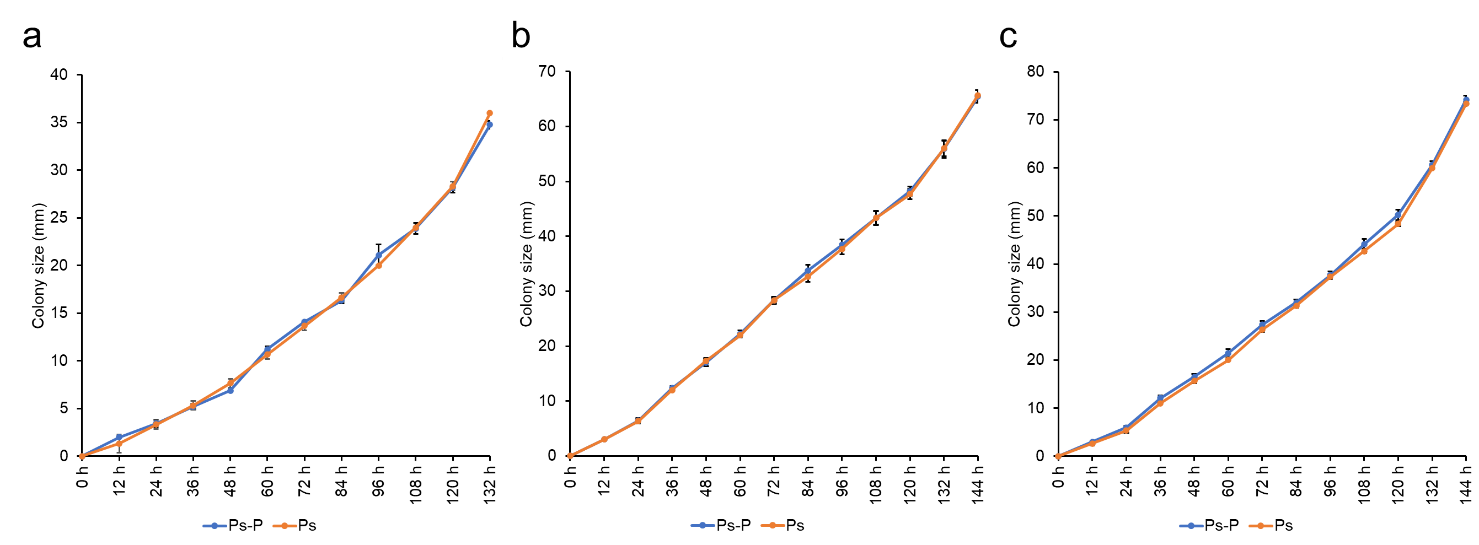


Figure S5. Comparison of colony diameter between the parental strain (Ps) and the isogenic strains without AsuPV1 (Ps-P) grown on (a) minimal agar medium containing 2% sucrose (MMS2A), (b) YPS2A, and (c) PDA. Three independently obtained strains of Ps-P were used. Each strain was cultured in triplicate. Error bars indicate standard deviation; the difference was tested using Welch’s *t*-test.


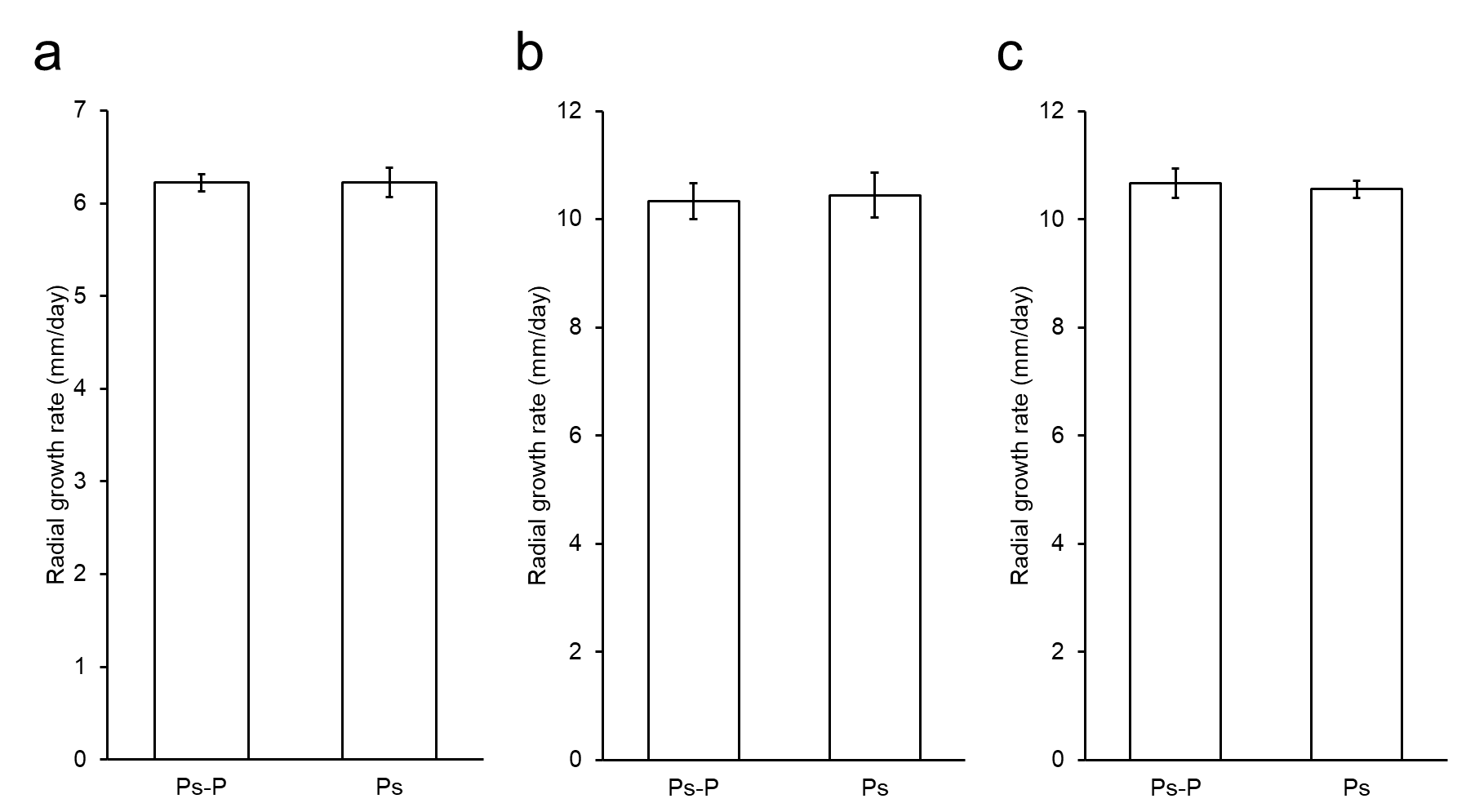


Figure S6. Comparison of average radial growth rates between Ps and Ps-P grown on (a) MMS2A, (b) YPS2A, and (c) PDA. Three independently obtained strains of Ps-P were used. Each strain was cultured in triplicate. Error bars indicate standard deviation; the difference was tested using Welch’s *t*-test.


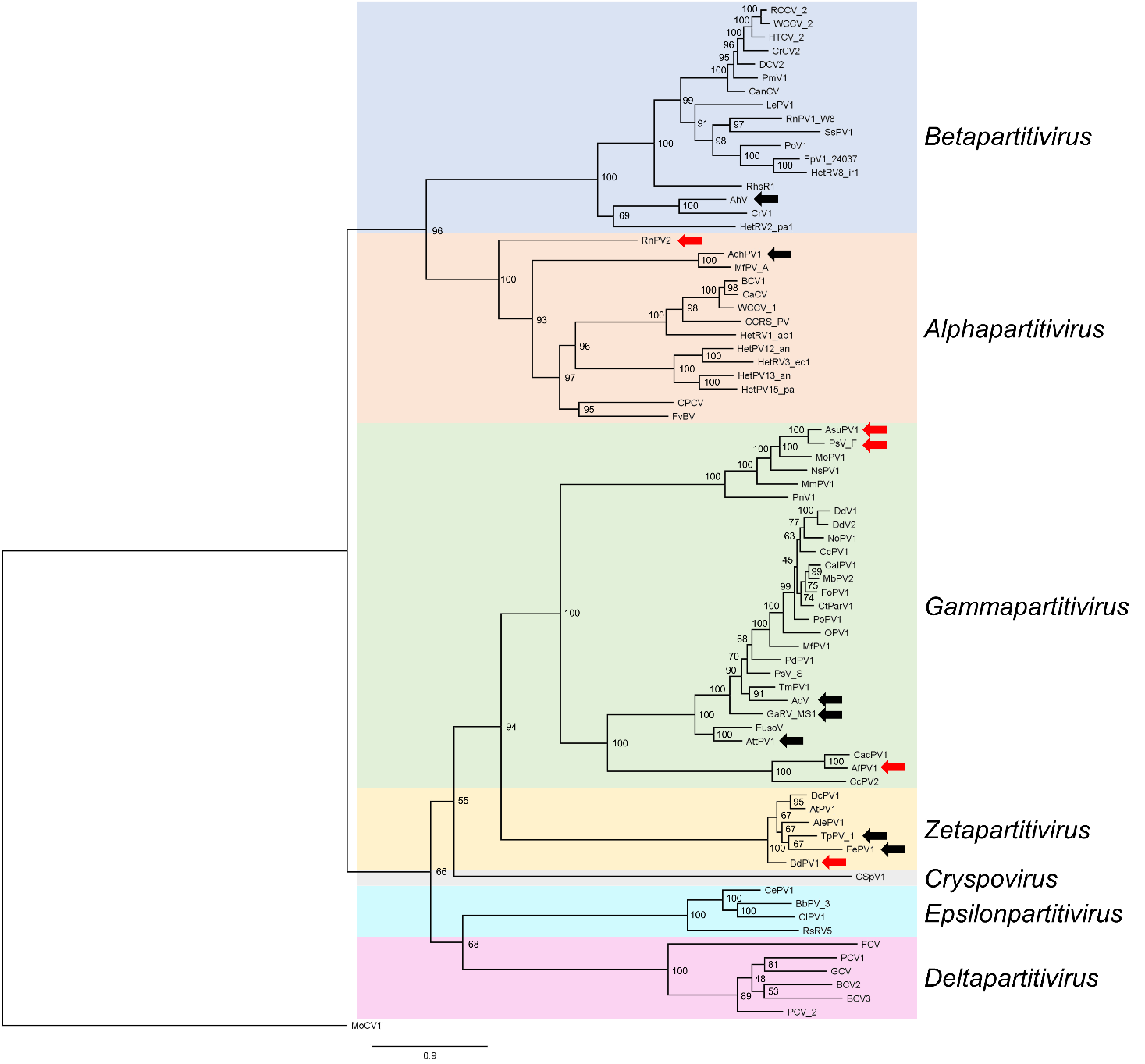


Figure S7. A maximum-likelihood tree of selected members of the family *Partitiviridae* based on RNA-dependent RNA polymerase (RdRP) amino acid sequences. The black or red arrows show viruses with tri-segmented genomes. The other partitiviruses in the tree have bi-segmented genomes except for MfPV-A, whose genomic organization is unknown. The red arrows indicate that encapsidation of each genomic segment of the pointed virus was experimentally confirmed. The numbers on the branches are the bootstrap percentages (1000 replicates). MoCV1 from the family *Chrysoviridae* is an outgroup. The full names and accession numbers of the viruses used to build the tree are shown in Table S1.

Table S1. List of partitiviruses used for phylogenetic analyses.

| Abbreviation | Name | Accession number |
| --- | --- | --- |
| AchPV1 | Aspergillus chevalieri partitivirus 1 | BFP29928 |
| AfPV1 | Aspergillus flavus partitivirus 1 | BED98276 |
| AhV | Atkinsonella hypoxylon virus | NP_604475 |
| AlePV1 | Aspergillus lentulus partitivirus 1 | BCH36641 |
| AoV | Aspergillus ochraceus virus | ABC86749 |
| AsuPV1 | Aspergillus sulphureus partitivirus 1 | BFP29933 |
| AtPV1 | Alternaria alternata partitivirus 1 | APT70073 |
| AttPV1 | Alternaria tenuissima partitivirus 1 | QTW52495 |
| BbPV-3 | Beauveria bassiana partitivirus 3 | QFP40245 |
| BCV1 | Beet cryptic virus 1 | YP_002308574 |
| BCV2 | Beet cryptic virus 2 | YP_009508068 |
| BCV3 | Beet cryptic virus 3 | YP_009665971 |
| BdPV1 | Botryosphaeria dothidea partitivirus 1 | AGZ84316 |
| CacPV1 | Colletotrichum acutatum partitivirus 1 | AGL42312 |
| CaCV | Carrot cryptic virus | YP_009508046 |
| CalPV1 | Colletotrichum alienum partitivirus 1 | WNA08356 |
| CanCV | Cannabis cryptic virus | YP_009293586 |
| CcPV1 | Cladosporium cladosporioides partitivirus 1 | WEU80701 |
| CcPV2 | Cladosporium cladosporioides partitivirus 2 | WEU80703 |
| CCRS-PV | Cherry chlorotic rusty spot associated partitivirus | CAH03668 |
| CePV1 | Colletotrichum eremochloae partitivirus 1 | AZT88590 |
| ClPV1 | Colletotrichum liriopes partitivirus 1 | QZB49018.1 |
| CPCV | Chondrostereum purpureum cryptic virus 1 | YP_009508236 |
| CrCV2 | Crimson clover cryptic virus 2 | YP_009508059 |
| CrV1 | Ceratocystis resinifera virus 1 | YP_001936016 |
| CSpV1 | Cryptosporidium parvum virus 1 | O15925 |
| CtParV1 | Colletotrichum truncatum partitivirus 1 | ALF46547 |
| DcPV1 | Delitschia confertaspora partitivirus 1 | AZT88584 |
| DCV2 | Dill cryptic virus 2 | YP_007891054 |
| DdV1 | Discula destructiva virus 1 | NP_116716 |
| DdV2 | Discula destructiva virus 2 | NP_620301 |
| FCV | Fig cryptic virus | YP_004429258 |
| FePV1 | Fusarium equiseti partitivirus 1 | QOW77954 |
| FoPV1 | Fusarium oxysporum partitivirus 1 | WJN00260.1 |
| FpV1-240374 | Fusarium poae virus 1-240374 | YP_009272951 |
| FusoV | Fusarium solani virus 1 | NP_624350 |
| FvBV | Flammulina velutipes browning virus | YP_009508048 |
| GaRV-MS1 | Gremmeniella abietina RNA virus MS1 | NP_659027 |
| GCV | Galphimia cryptic virus | WEU67078 |
| HetPV12-an1 | Heterobasidion partitivirus 12, strain 1 from Heterobasidion annosum | YP_009508051 |
| HetPV13-an1 | Heterobasidion partitivirus 13, strain 1 from Heterobasidion annosum | YP_009508053 |
| HetPV15-pa1 | Heterobasidion partitivirus 15, strain 1 from Heterobasidion parviporum | YP_009508056 |
| HetRV1-ab1 | Heterobasidion RNA virus 1, strain 1 from Heterobasidion abietinum | YP_009508049 |
| HetRV2-pa1 | Heterobasidion RNA virus 2, strain 1 from Heterobasidion parviporum | YP_009508061 |
| HetRV3-ec1 | Heterobasidion RNA virus 3, strain 1 from Heterobasidion ecrustosum | YP_009508058 |
| HetRV8-ir1 | Heterobasidion RNA virus 8, strain 1 from Heterobasidion irregulare | YP_009508063 |
| HTCV-2 | Hop trefoil cryptic virus 2 | YP_007889825 |
| LePV1 | Lentinula edodes partitivirus 1 | AQS27950 |
| MbPV2 | Metarhizium brunneum partitivirus 2 | QTC11257 |
| MfPV1 | Metarhizium flavoviride partitivirus 1 | AZU96334 |
| MfPV-A | Monilinia partitivirus A | QED42950 |
| MmPV1 | Metarhizium majus partitivirus 1 | UPO93685.1 |
| MoCV1 | Magnaporthe oryzae chrysovirus 1 | BBG92292 |
| MoPV1 | Magnaporthe oryzae partitivirus 1 | APP18151 |
| NoPV1 | Nigrospora oryzae partitivirus 1 | AXF84302 |
| NsPV1 | Nigrospora sphaerica partitivirus 1 | UBK24765 |
| OPV1 | Ophiostoma partitivirus 1 | YP_009508238 |
| PCV-1 | Pepper cryptic virus 1 | YP_009466859 |
| PCV-2 | Pepper cryptic virus 2 | YP_009351838 |
| PdPV1 | Pseudogymnoascus destructans partitivirus 1 | APG38312 |
| PmV1 | Primula malacoides virus 1 | YP_003104768 |
| PnV1 | Pythium nunn virus 1 | YP_009551507 |
| PoPV1 | Penicillium oxalicum partitivirus 1 | WPA89351 |
| PoV1 | Pleurotus ostreatus virus 1 | YP_227355 |
| PsV-F | Penicillium stoloniferum virus F | YP_271922 |
| PsV-S | Penicillium stoloniferum virus S | YP_052856 |
| RCCV-2 | Red clover cryptic virus 2 | YP_007889823 |
| RhsR1 | Rhizoctonia solani virus 717 | NP_620659 |
| RnPV1-W8 | Rosellinia necatrix partitivirus 1-W8 | YP_392480 |
| RnPV2 | Rosellinia necatrix partitivirus 2 | YP_007419077 |
| RsRV5 | Rhizoctonia solani dsRNA virus 5 | AVP26802 |
| SsPV1 | Sclerotinia sclerotiorum partitivirus 1 | AFR78160 |
| TmPV1 | Talaromyces marneffei partitivirus 1, isolate Q5 | AKF14166 |
| TpPV-1 | Talaromyces pinophilus partitivirus 1 | BEI31890 |
| WCCV-1 | White clover cryptic virus 1 | YP_086754 |
| WCCV-2 | White clover cryptic virus 2 | YP_007889821 |

Table S2. Pairwise identities of the third genomic segments of gammapartitiviruses with a tri-segmented genome.

|  | AsuPV1 | PsV-F | AttPV1 | AoV | GaRV-MS1 | AfPV1 |
| --- | --- | --- | --- | --- | --- | --- |
| AsuPV1 |  |  |  |  |  |  |
| PsV-F | 38.66% |  |  |  |  |  |
| AttPV1 | 21.25% | 33.15% |  |  |  |  |
| AoV | 23.97% | 21.03% | 19.49% |  |  |  |
| GaRV-MS1 | 21.03% | 25.51% | 22.78% | 33.31% |  |  |
| AfPV1 | 22.05% | 25.00% | 20.19% | 28.76% | 27.08% |  |

Table S3. Minimum inhibitory concentrations of 2'-*C*-methylcytidine (2CMC) and ribavirin. Yeast extract-peptone-2% sucrose medium (YPS2; 1% yeast extract, 2 % peptone, 2% sucrose) or potato dextrose broth (PDB) was used as the culture medium. Conidia or protoplasts of *A*. *sulphureus* NBRC4095 were used for inoculation.

|  |  | Conidia | Protoplasts |
| --- | --- | --- | --- |
| YPS2 | 2CMC | > 2 mg/mL | > 2 mg/mL |
|  | Ribavirin | > 2 mg/mL | > 2 mg/mL |
|  | Cycloheximide | 25 μg/mL | 50 μg/mL |
| PDB | 2CMC | > 2 mg/mL | > 2 mg/mL |
|  | Ribavirin | > 2 mg/mL | > 2 mg/mL |
|  | Cycloheximide | 50 μg/mL | > 100 μg/mL |

**Supplementary Methods**

Preparation of protoplasts

*A*. *sulphureus* NBRC4095 was cultured in potato dextrose broth (PDB) at 26.5 °C with agitation at 150 rpm for two days. Mycelia and culture supernatant were separated by filtration using Prowipe (Daio Paper Corporation, Tokyo, Japan). The mycelia were then incubated in protoplasting solution (10 mM sodium phosphate buffer (pH 6.0), 0.8 M NaCl, 5 mg/mL Yatalase (Takara Bio Inc., Shiga, Japan) with agitation at 60 rpm for 2 hours at 30 °C. The suspension was filtered through a cell strainer, pluriStrainer 40 μm (pluriSelect Life Science, Leipzig, Germany). The protoplasts were collected by centrifugation (1,000 *g*, 5 min), washed with 0.8 M NaCl twice, and then suspended in 0.8 M NaCl. The density of protoplasts in the suspension was determined using an improved Neubauer's counting chamber (Watson, Tokyo, Japan).

Minimum inhibitory concentration (MIC) test using conidia

MIC tests were performed using 96-well plates (stem, Tokyo, Japan), in which each well contained 200 μL of yeast extract-peptone-2% sucrose medium (YPS2; 1% yeast extract, 2 % peptone, 2% sucrose) or PDB. The concentration of 2'-*C*-methylcytidine (2CMC) and ribavirin was 2 mg/mL to 980 ng/mL (by serial dilution). Dimethyl sulfoxide and cycloheximide were used as a negative and a positive control, respectively. The concentration of cycloheximide was 100 μg/mL to 49 ng/mL (by serial dilution). Conidia of *A*. *sulphureus* NBRC4095 were inoculated at the density of 1.0 × 10^4^/mL, and incubated for 2 days at 26.5 °C. The assays were performed in triplicate.

MIC test using protoplasts

MIC tests were performed using 96-well plates (stem), in which each well contained 200 μL of YPS2 supplemented with 0.8M NaCl or PDB supplemented with 0.8M NaCl. The concentration of 2CMC and ribavirin was 2 mg/mL to 980 ng/mL (by serial dilution). Dimethyl sulfoxide and cycloheximide were used as a negative and a positive control, respectively. The concentration of cycloheximide was 100 μg/mL to 49 ng/mL (by serial dilution). Protoplasts of *A*. *sulphureus* NBRC4095 were inoculated at the density of 1.0 × 10^5^/mL, and incubated for 3 days at 26.5 °C. The assays were performed in triplicate.
